# Blood Eosinophil Levels in Newborns with Severe Indirect Hyperbilirubinemia Treated with Phototherapy

**Published:** 2014-06

**Authors:** Banu Aydın, Serdar Beken, Ayşegül Zenciroğlu, Dilek Dilli, Nurullah Okumuş

**Affiliations:** Neonatal Intensive Care Unit, Dr. Sami Ulus Maternity and Children Training and Research Hospital, Ankara, Turkey

**Keywords:** Newborn; Jaundice; Phototheraphy; Eosinophil; Asthma

## Abstract

***Objective:*** Newborns who suffer from jaundice and/or receive phototherapy (PT) are at a higher risk of developing asthma. In this study we aimed to investigate the relationship between bilirubin and peripheral eosinophil counts in newborns with severe hyperbilirubinemia needing PT.

***Methods:*** In this study, a retrospective analysis was performed on 306 newborns with severe hyperbilirubinemia with gestational age ≥35 weeks (Group 1) and the control group consisted of 295 age and gender-matched newborns (Group 2). Total serum bilirubin, hemoglobin, albumin, leucocyte and eosinophil counts before and after PT were recorded from medical charts.

***Findings*** ***:*** All the patients in Group 1 received phototherapy and 77 (25.2%) of them needed exchange transfusion (ET). Before receiving PT, the patients in Group 1 had lower levels of Hb and higher levels of total serum bilirubin and lymphocytes than those in Group 2 although there was no statistically significant difference with regard to peripheral eosinophil counts. Eosinophils were detected to be numerically lower in Group 1. Higher bilirubin subgroups had also lower eosinophil counts. The patients in Group 1 had lower levels of Hb, leucocyte, albumin and higher levels of eosinophil following PT.

***Conclusion:*** Peripheral eosinophil count may be affected by bilirubin levels and/or phototherapy. There is a need for further clinical research based on different models.

## Introduction

Indirect hyperbilirubinemia (IHB) is the most common cause of jaundice in the neonatal period^[^^1^^]^. Eosinophils are white blood cells of the immune system which are responsible for parasite defense reactions, allergic response, tissue inflammation, and immune modulation^[^^2^^]^. Eosinophils have a central role in the pathogenesis of asthma, therefore can be selectively targeted with therapeutic agents, which seems to provide clinical benefit in asthmatics patients with persistent airways eosinophilia^[^^3^^]^.

 Bilirubin is one of the end products of heme catabolism mediated by heme oxygenase and biliverdin reductase^[^^4^^]^. Even though bilirubin is known to have toxic effects, it also has antioxidant properties and protects the lungs and even the intestines^[^^5^^]^. Bilirubin was shown to inhibit vascular cell adhesion molecule-1 (VCAM-1)-dependent mechanisms^[^^6^^]^. VCAM-1 plays a major role in inflammatory cell migration and located on endothelial cell surface starts the adhesion of lymphocytes and eosinophils to the vascular endothelium^[^^7^^]^. Keshavan et al utilized a mouse model of airway inflammation similar to asthma, and they discovered VCAM-1-mediated pulmonary eosinophilia, lymphocytosis; and anti-inflammatory effects of bilirubin in the lung tissue and eosinophilia in circulation of these mice^[^^8^^]^. 

 It was reported that the newborns with jaundice requiring or not requiring PT are at greater risk of developing asthma later in life compared to newborns who do not suffer from jaundice^[^^9^^-^^11^^]^. To the best of our knowledge, there is no study in the literature that focuses on relationship between peripheral eosinophil counts and hyperbilirubinemia in jaundiced newborns who receive or not receive PT. Therefore, in this study we aimed to investigate the relationship between bilirubin and eosinophil counts in newborns with severe hyperbilirubinemia, and we also compared eosinophil counts variability before and after PT.

## Subjects and Methods

A retrospective analysis was performed on 306 newborns with severe hyperbilirubinemia who were admitted to the intensive care unit (NICU) at Dr Sami Ulus Maternity and Children Research and Training Hospital, a level III neonatal center in Ankara, Turkey; between January 2011 and December 2012. Severe hyperbilirubinemia was defined as having a total serum bilirubin (TSB) level ≥20 mg/dL and/or requiring FT or exchange transfusion (ET) according to the American Academy of Pediatrics (AAP) guidelines^[^^12^^]^. The study was approved by the Ethics Committee of the Keçiören Ethic Committee, Ankara, Turkey.

 The newborns with severe hyperbilirubinemia were assigned as study patients (Group 1) and treated according to the AAP guidelines. 295 age and gender-matched newborns that were checked for bilirubin levels and did not receive PT and diagnosed as physiological jaundice were analyzed as control group (Group 2).

*Inclusion criteria for the patient group:* Infants with a gestational age ≥35 weeks, TSB levels ≥20 mg/dL, and with no other accompanying pathologies than jaundice were included. 

*Exclusion criteria for the patient group* ***:*** Infants with a gestational age <35, TSB levels <20 mg/dL and other accompanying pathologies than jaundice were excluded.

*Inclusion criteria for the control group: *Infants with a gestational age ≥35 weeks and TSB levels of 10-15 mg/dL. 

*Exclusion criteria for the control group:* Infants with a gestational age <35 and TSB levels <10 mg/dL were excluded.

 The patients in Group 1 were also stratified depending on their TSB levels: Group A consisted of patients with a TSB level of 20-22.9 mg/dL, Group B consisted of patients with a TSB level of 23-24.9 mg/dL, and Group C consisted of patients with a TSB level ≥25 mg/dL.

 All patients' gender, gestational age, birth weight, type of delivery, and birth weight percentiles as well as the age of their mothers, the existence of preeclampsia and gestational diabetes in the mothers, whether the mothers had a history of smoking and the presence of allergic diseases in the family were taken from the medical records. 

 Depending on hyperbilirubinemia protocol of our unit, before PT treatment was commenced, 2 mL of blood sample was taken from all patients and analyzed for hemoglobin (Hb), leucocyte, lymphocyte, eosinophil, total and direct bilirubin, and albumin levels. When the TSB level fell 2 mg/dl below the PT threshold for patient's age, the treatment was terminated. Subsequently, 12-24 hours later, blood samples were obtained and re-analyzed for Hb, leucocyte, lymphocyte, eosinophil, TSB, and albumin.

 The patients in the control group, underwent the transcutaneous bilirubin control, and blood samples were obtained in those who had a bilirubin level of 10-15 mg/dL. The samples were analyzed for Hb, leucocyte, lymphocyte, eosinophil, and TSB. The patients who had a TSB level of 10-15 mg/dL were allocated to the control group. 

 Eosinophil count was determined via complete blood count test (Abx-Pentra120, France), using impedance and cytochemistry method. Biochemical analyses including bilirubin, and albumin were studied with spectrophotometric method (Beckman Coulter, DxC-8000; CA, USA). Transcutaneous bilirubin levels were checked with Drager Jaundice Meter (JM 103, Germany).

 In statistical analysis of the data, the software package called SPSS (version 16.0) was used. The Kolmogorov-Smirnov test was conducted to determine the shapes of the distribution of variables.

**Table 1:** Demographic features of the patients

**Parameter**	**Group 1 (n=306)**	**Group 2 (n=295)**	***P. value***
**Gender (Male/Female) n**	163/143	163/132	0.684
**Birth weight (grams)**	3149(468)	3185(443)	0.117
**Gestational age (weeks)**	39 (35-41)	39 (36-41)	0.08
**Age at admission (days)**	5 (1-12)	5 (2-10)	0.162
**Maternal age (years)**	26.5(5.2)	26.1(5.4)	0.922
**Gestationel diabetes n (%)**	10 (3.3)	10 (3.5)	>0.05
**Preeclampsia n (%)**	8 (2.6)	8 (2.9)	>0.05
**Smoking during pregnancy n (%)**	8 (2.6)	8 (2.7)	>0.05
**Family history of allergic disorders n (%)**	13 (4.2)	11 (4.0)	>0.05

In summarizing the data, mean±SD or median values (interquartile range, IQR) were used, as appropriate. Pearson or Spearman test was used for correlation analysis. Group comparisons were analyzed by Student *t*-test or Mann-Whitney U-test. Paired test or Wilcoxon test was used for paired samples. One-way ANOVA was used to compare three groups. A two-tailed *P-*value <0.05 was considered statistically significant.

***Findings***

A total of 306 cases were included in the patient group; 163 (53.3%) of these patients were males and 228 (74.5%) of the patients were born via spontaneous vaginal delivery. The patients were born at a median gestational age of 39 (35-41) weeks with a mean birth weight of 3149±468 gram. No statistically significant difference with regard to socio-demographic differences was observed between Group 1 and Group 2 (Table 1). 

The patients in Group 1 were hospitalized on postnatal day 5 (1-12). No underlying causes of jaundice could be determined in 74 (24.2%) of the patients. And the most common cause of jaundice was ABO incompatibility (n=88, 28.7%). As for all the patients in Group 2, the underlying cause of jaundice was physiological jaundice and none of them needed hospitalization. 

 All the patients in Group 1 received PT (2.8±1.6 days) and 77 (25.2%) of the patients needed ET. Blood tests revealed that before receiving PT, the patients in Group 1 had lower levels of Hb (*P*=0.001) and higher levels of TSB and lymphocytes than those in Group 2 (*P*=0.001, 0.004, respectively). Although with regard to eosinophils no statistically significant difference was observed, the levels of eosinophil were detected to be numerically lower in Group 1 (*P*=0.05) (Table 2). The patients in Group 1 (patients who received ET were excluded from this analysis, n=229) had lower levels of Hb, leucocyte, and albumin; and higher levels of eosinophil following the PT (*P*=0.001) (Table 3). 

 In Group 1, there was a negative correlation between TSB and lymphocyte levels (r= -0.182, *P*=0.001) and between TSB and eosinophil levels (r=-0.12, *P*=0.03), and a positive correlation between TSB and albumin (r= 0.23, *P*<0.001) before the patients received PT. 

**Table 2:** Comparison of Group 1 and 2 including total serum bilirubin, hemoglobin, leucocytes, lymphocytes and eosinophils

	**Group 1 (n=306)**	**Group 2 (n=295)**	***P. value***
**Total serum bilirubin** ** (mg/dl)**	23.4 (4.0)	12.8 (1.5)	0.001
**Hemoglobin** ** (g/dl)**	15.6 (2.8)	17.3 (2.0)	0.001
**Leucocyte** **s (/mm** ^3^ **)**	12675 (6729)	12457 (4417)	0.8
**Lymphocytes** ** (/mm** ^3^ **)**	5124.3 (3792.0)	4227 (1627)	0.004
**Eosinophils (/mm** ^3^ **)**	407.2 (243.7)	453 (208)	0.05

**Table 3:** Comparison of total serum bilirubin, hemoglobin, leucocytes, lymphocytes, eosinophils and albumin in Group 1 before and after phototheraphy

**Parameter**	**Before PT (n=229)**	**After PT (n=229)**	***P. value***
**Total serum bilirubin** ** (mg/dl)**	23.4 (4.0)	10.0 (2.2)	0.001
**Hemoglobin** ** (g/dl)**	15.6 (2.8)	13.8 (2.4)	0.001
**Leucocyte** **s (/mm** ^3^ **)**	12675 (6729)	11040 (3640)	0.001
**Lymphocytes** ** (/mm** ^3^ **)**	5124.3 (3792.0)	4953.7 (2026.2)	0.507
**Eosinophils** ** (/mm** ^3^ **)**	407.2 (243.7)	506.4 (357.8)	0.001
**Albumin (g/dl)**	3.2 (0.4)	3.0 (0.4)	0.001

As for the relationship between the blood values obtained after the patients in Group 1 received PT, a positive correlation between TSB and eosinophil (r=0.15, *P*=0.02) was found.

**Fig. 1:**
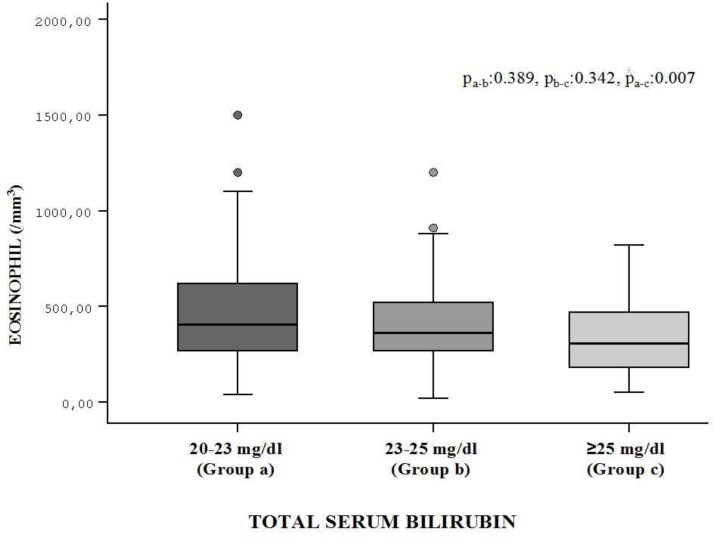
Comparison of eosinophil levels in group I (group A, group B and group C)

 The patients in Group 1 were stratified depending on their TSB levels: (Group A; 20-22.9 mg/dL, Group B; 23-25 mg/dL, and Group C; >25 mg/dL). No statistically significant difference with regard to socio-demographic differences was observed within these subgroups (data not given, *P*>0.05). The assessment of the Hb, leukocyte, lymphocyte, eosinophil and albumin levels obtained before PT revealed that there was a difference in these three groups with regard to eosinophil. Group A had higher eosinophil levels compared to Group C (F=4.65, *P*=0.01) (Table 4, Fig. 1).

## Discussion

In our study, we found that the newborns who suffered from severe hyperbilirubinemia before the commencement of PT had a significantly higher lymphocyte count than those who suffered from a much less severe hyperbilirubinemia (TSB 10-15 mg/dL), and newborns with severe IHB were observed to have lower levels of eosinophils, even though this difference was not statistically significant. In term neonates, the mean value for eosinophils is reported to be 550/uL, ranged between 140 to 1300/uL^[^^13^^]^. Although patient’s eosinophil levels are within normal range, we showed that higher bilirubin subgroups had lower eosinophil counts. 

**Table 4:** Comparison of hemoglobin, leucocytes, lymphocytes, eosinophils and albumin in Group 1 subgroups depending on bilirubin levels before phototherapy

**TSB Levels**	**20-22.9 mg/dl** **(Group A)** **[n=151 (49.3%)]**	**23-25 mg/dl** **(Group B)** **[n=72 (23.5%)]**	**>25 mg/dl** **(Group C)** **[n=83 (27.2%)]**	**F**	***P. value***	***P*** **. value**
**Hemoglobin** **(g/dl)**	16.0 (2.7)	16.2 (2.6)	15.7 (2.4)	0.464	0.6	a-b:0.871 b-c:0.604 a-c:0.816
**Leucocyte** **s** **(/mm** ^3^ **)**	11663 (5033)	11826 (3339)	11708 (4073)	0.031	1	a-b:0.966 b-c:0.986 a-c:0.997
**Lymphocytes** **(/mm** ^3^ **)**	5118 (2950)	4873 (2042)	4543 (1828)	1.373	0.3	a-b:0.781 b-c:0.703 a-c:0.225
**Eosinophil** **s** **(/mm** ^3^ **)**	463 (265)	414 (225)	356 (251)	4.648	0.01	a-b:0.389 b-c:0.342 a-c:0.007
**Albumin** **(g/dl)**	3.2(0.3)	3.2(0.3)	3.2 (0.3)	1.709	0.2	a-b:0.640 b-c:0.734 a-c:0.163

* Tukey HSD

It has been proven in the limited number of animal experiments that in mouse model of asthma, the mice that are given bilirubin have low levels of lymphocytes and eosinophils in the lung tissue, but also that their blood eosinophil levels increase para-doxically^[^^8^^,^^14^^,^^15^^]^. However, no clinical trials have yet been conducted to observe the effects of bilirubin on human beings. Nevertheless, based on the animal experiments, it might be claimed that the suppression of VCAM-1 accounts for the low levels of blood eosinophil in our study. The fact that TSB levels are in negative correlation with eosinophil levels supports our claim. Paradoxically, it can also be claimed that high levels of TSB have a serious neurotoxic effect on one hand, and on the other, they might have a protective effect against the development of allergic diseases later in life. But the mechanism underlying the high levels of lymphocytes is not clearly known. 

 High levels of bilirubin may induce a decrease in eosinophil levels by suppressing VCAM-1, so that, any treatment that aims to decrease TSB levels may be expected to increase the levels of eosinophils. The most commonly used method in the treatment of IHB is PT. Recent studies have shown that certain cytokines released by peripheral blood mononuclear cells are affected in full-term neonates who are given PT to treat hyperbilirubinemia. Sirota et al pointed out in their study on newborns who receive PT that there was a significant increase in the release of IL-2 and IL-10 and a decrease in the secretion of IL-1beta. They claimed that IL-6 and IL-10 are produced from keratinocytes in association with PT^[^^16^^]^. Kondo et al, on the other hand, who exposed normal cultured human epidermal keratinocytes to 10-300 J/m^2^ UVB irradiation, found that there was an increase in IL-8 mRNA level^[^^17^^]^. There is no study in the literature that focuses on how PT affects the level of eosinophils. However, the fact that notably IL-10 is a chemoattractant suggests that eosinophilia might develop in association with PT. In the light of all this data, it might be claimed that bilirubin is protective against allergic diseases and that PT might trigger allergic diseases. There are only 3 studies in the literature regarding this matter^[^^9^^-^^11^^]^. In their study, Aspberg et al reported that in the absence of other neonatal and maternal risk factors newborns who suffer from jaundice and/or who therefore receive PT, are at a 1.5 times higher risk of developing asthma later in life than newborns who do not suffer from jaundice^[^^10^^]^. The same researchers showed in another study that neonatal jaundice and/or exposure to PT is the single determinant of the risk of developing asthma after 12 years of age^[^^9^^]^. Ku et al reported that infants who were diagnosed with neonatal jaundice had 1.64 times higher risk for asthma^[^^11^^]^. However, in the mentioned studies, it is not clear whether the risk of developing asthma is associated with bilirubinemia or PT. In our study, we also found that the levels of serum albumin levels decreased following PT. In the light of literature and our data, one might suggest that not bilirubin but PT may cause a decrease in antioxidant capacity, and may be related risk of developing asthma Human serum albumin, which is a multifunctional, non-glycosylated, negatively charged transport protein in human blood plasma serving antioxidant and enzymatic functions, is the most important antioxidant in an individual who is exposed to oxidative stress^[^^18^^]^. In our study, we found that there is a positive relationship between levels of TSB and serum albumin. This brings to mind that bilirubin, which is known to be an antioxidant, can act like a pro-oxidant when found in large quantities. 

 The limitation of this study is being of retrospective nature and without randomization. The literature would benefit from prospective studies in which such a randomization measurement is made.

## Conclusion

Peripheral eosinophil count may be affected by bilirubin levels and/or phototherapy. The exact mechanism of neonatal jaundice/phototherapy and later risk of developing asthma is not clear yet, further prospective studies in larger groups are needed.

## Authors’ Contribution

B. Aydın: Study design, supervision and manuscript writing

S. Beken S: Data collection and analysis

D. Dilli, A. Zenciroğlu, N. Okumuş; Data collection. 

N. Okumuş: The guarantor

All authors approved the final version of the paper 
